# Combined Effects of Prone Positioning and Airway Pressure Release Ventilation on Oxygenation in Patients with COVID-19 ARDS

**DOI:** 10.4274/TJAR.2022.22783

**Published:** 2023-06-16

**Authors:** Bişar Ergün, Mehmet Nuri Yakar, Murat Küçük, Narmin Baghiyeva, Ahmet Naci Emecen, Erdem Yaka, Begüm Ergan, Ali Necati Gökmen

**Affiliations:** 1 Department of Internal Medicine and Critical Care, Dokuz Eylül University Faculty of Medicine, İzmir, Turkey; 2 Department of Anaesthesiology and Critical Care, Dokuz Eylül University Faculty of Medicine, İzmir, Turkey; 3 Department of Public Health, Epidemiology Subsection, Dokuz Eylül University Faculty of Medicine, İzmir, Turkey; 4 Department of Neurology and Critical Care, Dokuz Eylül University Faculty of Medicine, İzmir, Turkey; 5 Department of Pulmonary and Critical Care, Dokuz Eylül University Faculty of Medicine, İzmir, Turkey

**Keywords:** Airway pressure-release ventilation, ARDS, intensive care units, mortality, SARS-CoV-2

## Abstract

**Objective:**

Coronavirus disease 2019 (COVID-19) can cause acute respiratory distress syndrome (ARDS). Invasive mechanical ventilation (IMV) support and prone positioning are essential treatments for severe COVID-19 ARDS. We aimed to determine the combined effect of prone position and airway pressure release ventilation (APRV) modes on oxygen improvement in mechanically-ventilated patients with COVID-19.

**Methods:**

This prospective observational study included 40 eligible patients (13 female, 27 male). Of 40 patients, 23 (57.5%) were ventilated with APRV and 17 (42.5%) were ventilated with controlled modes. A prone position was applied when the PaO_2_/FiO_2_ ratio <150 mmHg despite IMV in COVID-19 ARDS. The numbers of patients who completed the first, second, and third prone were 40, 25, and 15, respectively. Incident barotrauma events were diagnosed by both clinical findings and radiological images.

**Results:**

After the second prone, the PaO_2_/FiO_2_ ratio of the APRV group was higher compared to the PaO_2_/FiO_2_ ratio of the control group [189 (150-237)] vs. 127 (100-146) mmHg, respectively, (*P*=0.025). Similarly, after the third prone, the PaO_2_/FiO_2_ ratio of the APRV group was higher compared to the PaO_2_/FiO_2_ ratio of the control group [194 (132-263)] vs. 83 (71-136) mmHg, respectively, (*P*=0.021). Barotrauma events were detected in 13.0% of the patients in the APRV group and 11.8% of the patients in the control group (*P*=1000). The 28-day mortality was not different in the APRV group than in the control group (73.9% vs. 70.6%, respectively, *P*=1000).

**Conclusion:**

Using the APRV mode during prone positioning improves oxygenation, especially in the second and third prone positions, without increasing the risk of barotrauma. However, no benefit on mortality was detected.

**Main Points**

• When combining prone positioning with airway pressure release ventilation (APRV), improvement in oxygenation is better than controlled mode, especially in the second and third prone positions.

• APRV can be safely used in Coronavirus disease-2019 (COVID-19) acute respiratory distress syndrome (ARDS) as barotrauma events are similar in both groups.

• APRV did not reduce mortality more than controlled modes in COVID-19 ARDS patients.

## Introduction

Coronavirus disease-2019 (COVID-19) is a pandemic caused by the severe acute respiratory syndrome. Coronavirus mainly affects the pulmonary system and can cause acute respiratory distress syndrome (ARDS).^1,2^ The incidence of severe ARDS was 35% in mechanically ventilated intensive care unit (ICU) patients.^3^ Mortality in COVID-19 patients with mild, moderate, and severe ARDS was 25, 33, and 41% respectively.^3^ The survival advantage of prone position among patients with severe ARDS has been demonstrated in meta-analysis and randomized trials for a long-time.^4,5^ In the supine position, since the dorsal trans-pulmonary pressure (airway opening pressure-pleural pressure) is higher than the ventral trans-pulmonary pressure, the ventral alveoli are prone to over-inflation and the dorsal alveoli are prone to atelectasis.^6^ In the prone position, the difference between the dorsal and ventral trans-pulmonary pressure decreases and results in more homogeneous ventilation, lung aeration, and strain distribution than in the supine position.^7^ Although ventilation distribution is affected by prone positioning, pulmonary perfusion is thought to be less affected by gravity.^8^ Providing a better ventilation-perfusion match results in improved gas exchange, and a homogeneous distribution of ventilation results in a reduced risk of ventilator-induced lung injury.^7,8,9^ Patients with COVID-19 ARDS have lung morphology and respiratory mechanics similar to patients with classical ARDS.^10^ Mechanically ventilated COVID-19 patients with refractory hypoxemia were administered the prone position for rescue therapy,^3,11,12^ resulting in improved oxygenation^3,11^ and increased survival.^12^

Airway pressure-release ventilation (APRV) is an inverse ratio, pressure controlled, time-cycled, intermittent mandatory ventilation.^13^ APRV delivers two levels of continuous positive airway pressure at which high pressure (P high) is delivered for a long duration (T high) and then falls to a lower pressure (P low) for a shorter duration (T low).^14^ Maintaining a constant airway pressure (P high) for a long time (T high) ensures that multiple alveolar units are recruited, resulting in a greater surface area for gas exchange.^15^ APRV permits spontaneous breaths at any time in the respiratory cycle.^13^ Spontaneous breathing may improve the redistribution of ventilation to dependent lung areas, provide better ventilation/perfusion matching, improve venous return, and reduce the need for sedation and neuromuscular blockade.^16^ APRV significantly increases the PaO_2_/FiO_2_ ratio and improves oxygenation in patients with ARDS compared with controlled methods.^17^

Overlapping physiological mechanisms that improve ventilation-perfusion mismatch in prone positioning and APRV may have potential synergistic effects on improving oxygenation in patients with COVID-19 ARDS. In this study, we evaluated the effects of combining APRV and prone positioning on gas exchange and mortality in patients with COVID-19 ARDS.

## Methods

### Study Population

After approval from the Local Ethics Committee of Dokuz Eylül University Non-Invasive Research Ethics Committee (date; 01.02.2021 and number; 2021/03-18) and the Turkish Ministry of Health, this prospective observational study was conducted in adult intensive care units of our center. All participants provided written informed consent. Between December 2020 and May 2021, all intubated patients (18 years and older) who met the Berlin criteria,^18^ received both pronation and APRV or controlled mode interventions, and those diagnosed with COVID-19 were included in the study. SARS-CoV-2 infection was confirmed by either using a reverse transcriptase-polymerase chain reaction (RT-PCR) tested on respiratory samples or with clinical characteristics, laboratory, and computed tomography findings. Patients who did not meet the Berlin criteria and did not take the prone position after invasive mechanical ventilation (IMV) interventions were excluded from the study.

### Definitions and Measurements

Our center uses APRV (Dräger Evita V300 and infinity V500, Lubeck, Germany) for patients with severe COVID-19-associated ARDS. APRV parameters were adjusted by an intensive care physician regarding previous guidelines.^15^ It was aimed to maintain spontaneous breathing in the APRV group and was continuously monitored. Patients in the control group were ventilated according to the ARDSNet protocol. In our center, we applied the prone position when the PaO_2_/FiO_2_ ratio <150 mmHg despite IMV in COVID-19 ARDS. Prone positioning was performed in normal ICU beds. Patients with hemodynamic instability did not receive the prone position. Data on ICU-acquired infections included ventilator-associated pneumonia, bloodstream infections, and urinary tract infections. Incident barotrauma events, including new subcutaneous emphysema, pneumomediastinum, pneumopericardium, or pneumothorax were diagnosed by both clinical findings and radiological images. Sedation depth was assessed using the Richmond Agitation-Sedation Scale (RASS).^19^ The sedation goal for most patients was a RASS score of -2 to +1.^19^ For patients requiring deeper levels of sedation in the prone position, the most comfortable level that preserves spontaneous breaths was aimed for.

### Variables

The demographic data (age, gender, smoking history, comorbidities), medical history, anthropometric measurements (Body Mass Index), Charlson Comorbidity Index (CCI), Acute Physiology and Chronic Health Evaluation (APACHE) II, and Sequential Organ Failure Assessment (SOFA) scores were recorded. Blood pressure records were obtained from the first measurement of ICU admission. Disease characteristics for COVID-19 including RT-PCR results and blood tests were collected. The parameters of the mechanical ventilation and of the arterial blood gas analysis were recorded an hour before turning the patient to the prone position and within an hour following the prone episode. Sedative, analgesic, and muscle relaxant drugs were recorded in the prone periods. Complications such as emphysema, pneumothorax, hypotension, need for vasopressors, cardiac arrhythmia, vascular access removal, intubation tube removal, pressure ulcers, airway obstruction, corneal abrasion, oliguria, and anuria were recorded during prone position. Major events during ICU stay [presence of septic shock, ICU acquired infections, AKI, renal replacement therapy (RRT)] were recorded. Lengths of ICU and hospital stays, and mortality was recorded.

### Outcomes

The primary outcome of the study was whether the combined use of APRV and prone positioning improves oxygenation in mechanically ventilated patients with severe COVID-19 ARDS. Secondary outcomes were the effects of the combined use of APRV and prone positioning on the length of stay and mortality.

### Statistical Analysis

All categorical variables were expressed as numbers and percentages, and continuous variables were expressed as median and interquartile ranges. Categorical variables between groups were compared with the chi-square or Fisher’s exact test, and continuous variables were compared with the Mann-Whitney U test. A two-tailed *P* value of <0.05 was considered statistically significant. Statistical analysis was performed using SPSS (Statistical Package for the Social Sciences Version 24, IBM Corp., Armonk, N.Y., USA).

## Results

### General Characteristics

**Figure 1:**
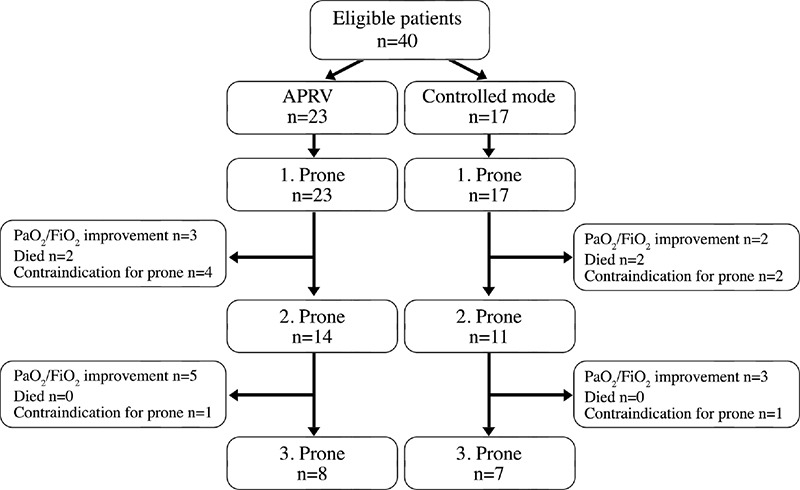
Flowchart of the study population. APRV, airway pressure release ventilation.

A total of 40 patients admitted to the ICU with COVID-19 were included in the study (Figure 1). All patients were mechanically ventilated and required at least one intervention of proning. The numbers of patients who completed the first, second, and third prone were 40, 25, and 15, respectively. Of the 40 patients, 27 (67.5%) were male and the median age of the study population was 65.0 (57.3-72.0 years; Table 1). A total of 23 patients were ventilated with APRV and 17 patients were ventilated with controlled modes. In the controlled mode group, 10 patients were ventilated in volume-controlled mode and 7 patients in pressure-controlled mode. Demographic factors, disease severity scores, and the PaO_2_/FiO_2_ ratio at admission and before intubation was similar in both groups. There was no difference between the groups in time from ICU admission to intubation and time from intubation to the onset of prone episodes. The time from ICU admission to intubation was 34.0 (6.0-99.0) hours. During this period before intubation, the most appropriate interventions including NIV, high-flow nasal oxygen, and awake-proning interventions were applied to the patients. After intubation, the duration of prone periods was similar between the two groups. D-dimer levels were lower in the APRV group than in the controlled mode group [1.37 (0.70-2.22)] vs. 3.60 (1.08-15.28) g mL^-1^, respectively, P=0.042). Other laboratory parameters were similar between the two groups.

### The Characteristics of the 1. Prone

Of the 40 patients in the first prone position, 23 were ventilated with APRV and 17 were ventilated with a controlled mode (Table 2).

In patients ventilated with APRV, the median (interquartile range) of the PaO_2_/FiO_2_ ratio before the first prone was not different when compared with patients ventilated with controlled modes [87 (73-113)] vs. 98 (82-119)] mmHg, respectively, *P*=0.193). After the first prone, the PaO_2_/FiO_2_ ratio of the APRV group was higher compared to the PaO_2_/FiO_2_ ratio of the controlled mode group, but it was not statistically significant [155 (125-185)] vs. 132 (110-150) mmHg, respectively, *P*=0.151).

### The Characteristics of the 2. Prone

Two patients in the APRV group and two patients in the controlled mode group died at follow-up after the first prone period. The physicians did not require the second prone position because the PaO_2_/FiO_2_ ratio improved for three patients in the APRV group and for two patients in the controlled mode group after the first prone. The second prone was not applied to four patients in the APRV group and to two patients in the controlled mode group because of hemodynamic instability. Of the 25 patients in the second prone position, 14 were ventilated with APRV and 11 were ventilated in the controlled mode.

Before the second prone, the PaO_2_/FiO_2_ ratio was 136 (96-171) mmHg in the APRV group and 123 (77-147) mmHg in the controlled mode group (*P*=0.149; Table 3). After the second prone, the PaO_2_/FiO_2_ ratio of the APRV group was higher compared to the PaO_2_/FiO_2_ ratio of the controlled mode group [189 (150-237)] vs. 127 (100-146) mmHg, respectively, *P*=0.025).

### Characteristics of the Third Prone

The physicians did not require the third prone position because the PaO_2_/FiO_2_ ratio improved following the second prone position for five patients in the APRV group and for three patients in the controlled mode group. The third prone was not applied to one patient in the APRV group and one patient in the controlled mode group due to hemodynamic instability. Of the 15 patients in the second prone position, 8 were ventilated with APRV and 7 were ventilated with the controlled mode.

**Figure 2:**
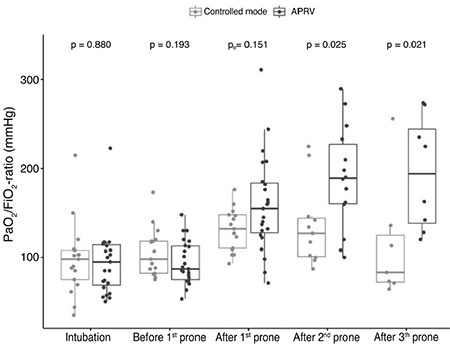
Median (interquartile range) of the PaO_2_/FiO_2_ ratio (mmHg) before the intubation and during the prone positioning in the study groups. APRV, airway pressure release ventilation; PaO_2_, partial pressure of arterial oxygen; FiO_2_, fraction of inspired oxygen.

Before the third prone, the PaO_2_/FiO_2_ ratio was 132 (81-177) mmHg in the APRV group and 95 (57-102) mmHg in the controlled mode group (*P*=0.024; Table 4). After the third prone, the PaO_2_/FiO_2_ ratio of the APRV group was higher compared to the PaO_2_/FiO_2_ ratio of the controlled mode group [194 (132-263)] vs. 83 (71-136) mmHg, respectively, *P*=0.021). The change in the PaO_2_/FiO_2_ ratio over time is presented in Figure 2.

### Major Events and Complications During the Prone Position and During ICU Stay

There was no difference between the two groups in major events or complications associated with prone positions (Table 5).

Spontaneous subcutaneous emphysema was detected in two patients in the pre-intubation follow-up. After intubation, one was ventilated with the controlled mode and the other with APRV. Emphysema did not worsen after IMV in both patients. Barotrauma events, including new subcutaneous emphysema, pneumomediastinum, pneumopericardium, or pneumothorax, were detected in 5 (12.5%) patients. One patient in the APRV group and one patient in the controlled mode group required a chest tube after barotrauma. The incidence of barotrauma events were not different in the APRV group and in the controlled mode group (13.0% vs. 11.8%, respectively; *P*=1000).

One of two patients with spontaneous subcutaneous emphysema survived. All 5 patients with barotrauma died.

### ICU Length of Stay and 28-day Mortality

The length of stay in the ICU was similar in both groups. The 28-day mortality was 73.9% in the APRV group and 70.6% in the controlled mode group (*P*=1000).

## Discussion

This prospective study addressed the possible combined effect of APRV and prone positioning on the improvement of oxygenation in patients with severe COVID-19, and obtained three important results. Firstly, when combining prone positioning with APRV, improvement in oxygenation was better than with the controlled mode, especially in the second and third prone positions. Secondly, APRV can be safely used in COVID-19 ARDS patients because barotrauma events are similar in both groups. Thirdly, APRV did not reduce mortality more than controlled modes in COVID-19 patients with ARDS.

To our knowledge, research on combined APRV and prone positioning is limited to one randomized clinical trial,^20^ and a retrospective study of patients with severe 2009 pandemic influenza A (H1N1) pneumonia.^21^ In the randomized controlled trial, 33 patients with acute lung injury who required the prone position were ventilated with either synchronized intermittant mandatory ventilation (SIMV) or APRV. They found that the PaO_2_/FiO_2_ ratio of the APRV group was greater than that of the SIMV group after the second prone [82 (37.0-141.0)] and 50 (24.0-68.0) mmHg, *P*=0.02, respectively. However, serious complications and 28-day mortality were similar in both groups in the randomized controlled trials.^20^ In a retrospective study of patients with ARDS associated with 2009 pandemic influenza A (H1N1), 11 of 14 mechanically ventilated patients had refractory hypoxemia despite APRV administration. Maintenance of APRV and following proving improved hypoxemia in these patients.^21^ Likewise, the positive effect of combined APRV ventilation and proning on the improvement of oxygenation have been demonstrated in a case series.^22^ Our findings were similar to the literature. In this study, after the first prone period, the PaO_2_/FiO_2_ ratio was higher in the APRV group than in the controlled mode group but was not statistically significant. After the second prone period, the PaO_2_/FiO_2_ ratio was significantly higher in the APRV group than in the controlled mode group, and this significance was maintained after the third prone position.

In a historical-comparative study, barotrauma was detected in 15% (n = 89) of 601 COVID-ARDS patients, while barotrauma was detected in 10% (n = 28) of 285 patients with non-COVID-ARDS in the same center in previous years.^23^ In another study of 20 mechanically ventilated patients with COVID-19, barotrauma events were detected in 8 (40%) patients.^24^ Not only the result of barotrauma but also spontaneous subcutaneous emphysema or pneumomediastinum/pneumothorax was detected in COVID-19 patients.^25,26^ High barotrauma events and cases of spontaneous pneumomediastinum/pneumothorax in COVID-19 patients raise questions about whether COVID-19 infection uniquely increases risk. In our study, we detected two patients with spontaneous subcutaneous emphysema at follow-up before IMV administration. Barotrauma events had a similar rate with literature in mechanically ventilated patients in our study. Barotrauma was an independent risk factor for death in mechanically ventilated COVID-19 patients.^23^ Similarly, in this study, all five patients with barotrauma died.

In a meta-analysis, including 57,420 adult patients with COVID-19 who received IMV, the overall reported case fatality rate (CFR) was estimated as 45% [95% confidence interval (CI), 39-52%].^27^ In this meta-analysis, among studies in which age-stratified CFR was available, pooled CFR estimates were 84.4% (95% CI, 83.3-85.4%) in patients with age above 80 years.^27^ In previous studies, high mortality rates were reported in patients undergoing IMV.^28^ Similarly, 28-day mortality was 72.5% (n = 29) in our specific study of patients with ARDS who underwent IMV and proning.

### Limitations and Strengths of the Study

The limitations of the study are as follows: (1) Although care was taken to maintain spontaneous breathing in the APRV group, in rare cases, patients required temporary deep sedation due to prone position intolerance; (2) We did not correlate plateau pressures between groups during prone positioning because it was not possible to measure in APRV ventilation; (3) The sample size was small. On the other hand, our study had several strengths. This study was conducted on a homogenous population that included patients with ARDS. The factors affecting oxygenation were similar in both groups. This homogeneity can make comparisons between groups more clear.

## Conclusion

Prone positioning and APRV ventilation have advantageous synergistic effects on oxygenation without increasing complications in patients with COVID-19 ARDS. This combination can be considered rescue therapy in refractory hypoxemia in this group of patients. However, improvement in oxygenation did not benefit mortality. The effect of APRV ventilation and proning on mortality in COVID-19 ARDS need to be investigated in larger studies.

## Figures and Tables

**Table 1:**
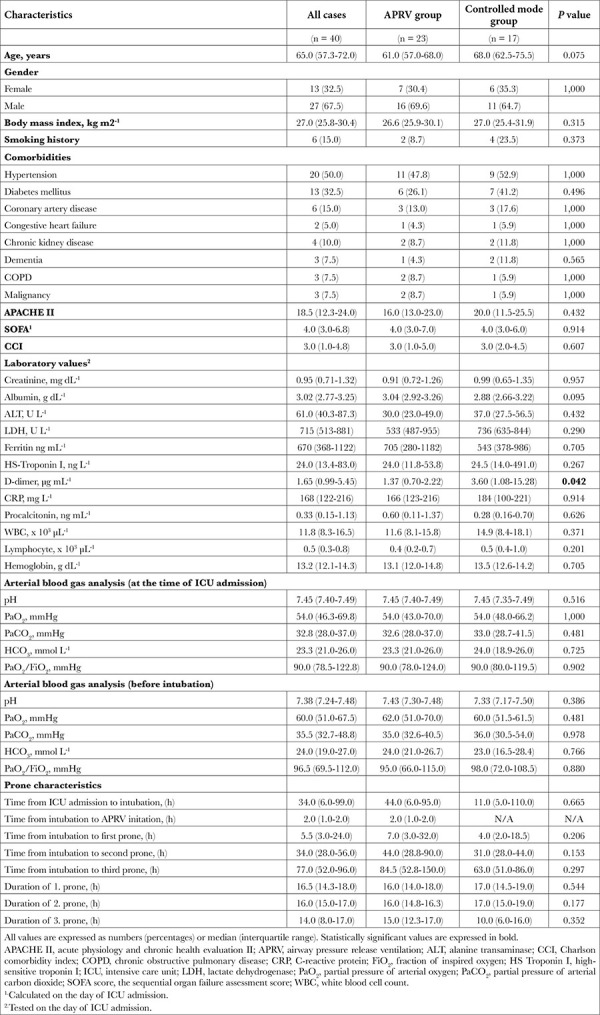
Demographic and Clinical Characteristics of Patients (Univariate Analysis)

**Table 2:**
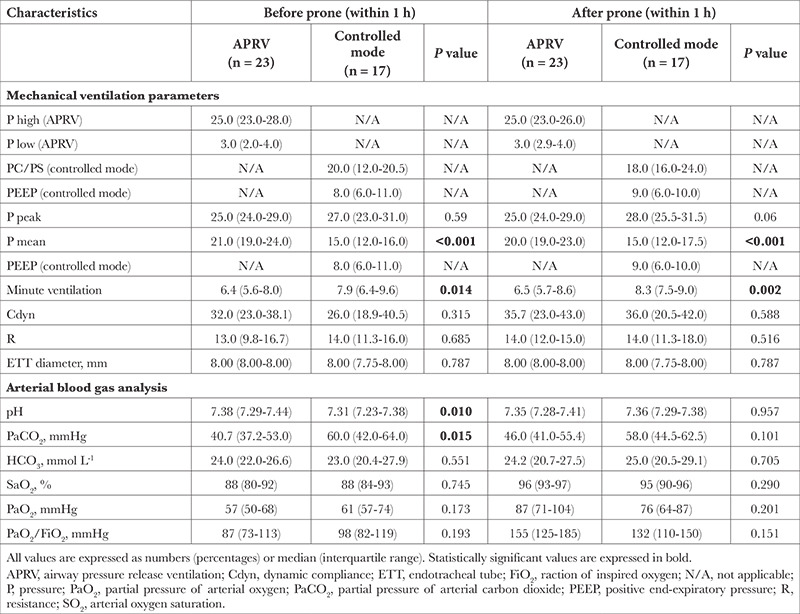
Characteristics Before and After 1. Prone Position

**Table 3:**
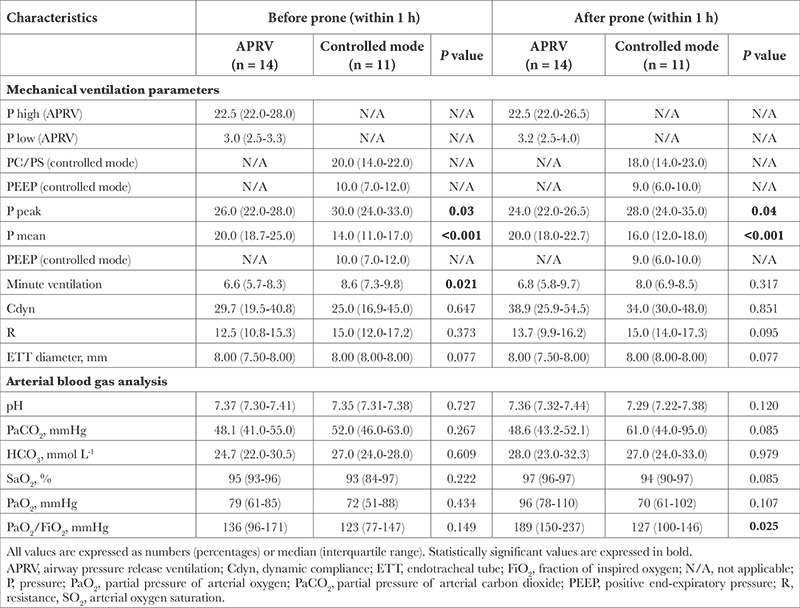
Characteristics Before and After 2. Prone Position

**Table 4:**
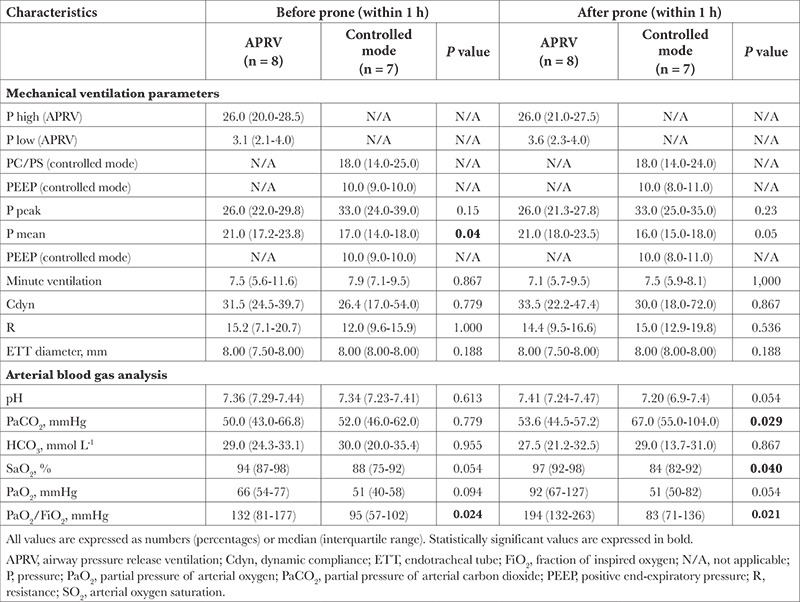
Characteristics Before and After 3. Prone Position

**Table 5:**
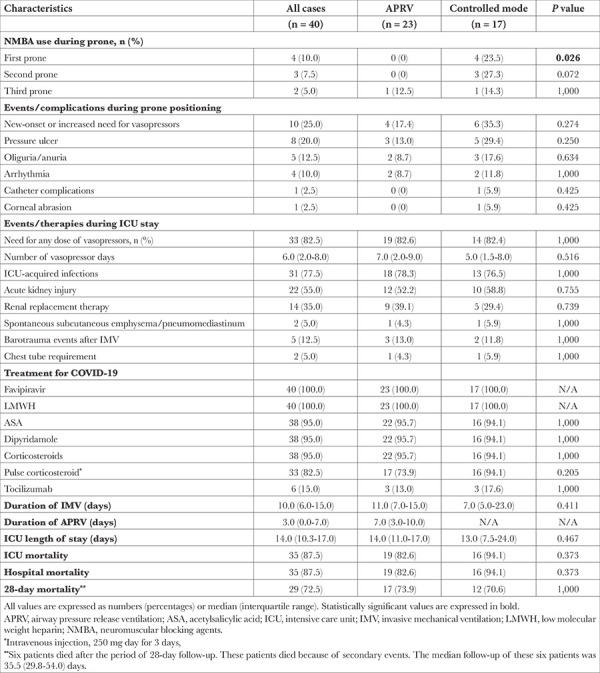
Outcomes (Univariate Analysis)
